# Application of Argon Ion Implantation to Improve the Surface Properties of Materials Based on PLA and Lignocellulosic Particles

**DOI:** 10.3390/molecules30091948

**Published:** 2025-04-28

**Authors:** Izabela Betlej, Marek Barlak, Karolina Lipska, Piotr Boruszewski, Piotr Borysiuk

**Affiliations:** 1 Institute of Wood Sciences and Furniture, Warsaw University of Life Sciences—SGGW, 159 Nowoursynowska St., 02-776 Warsaw, Poland; karolina_lipska@sggw.edu.pl (K.L.); piotr_boruszewski@sggw.edu.pl (P.B.); 2 Ion Beam Technology Division, Material Physics Department, National Centre for Nuclear Research Świerk, 7 Sołtana St., 05-400 Otwock, Poland; marek.barlak@ncbj.gov.pl

**Keywords:** ion implantation, wood–plastic composites, polylactic acid, wettability, color change

## Abstract

The new wood–plastic composites (WPC) biocomposites, a promising blend of poly(lactic acid) (PLA) and lignocellulosic fillers, are the subject of our study. We used bark and sawdust at 40, 50, and 60% as PLA fillers. The innovative use of ion implantation to modify the surface properties of the produced composites could have significant implications. Argon ions were used in three dosages (1 × 10^15^, 1 × 10^16^, and 1 × 10^17^ cm^−2^) at an accelerating voltage of 60 kV. The modified composites were then analyzed for changes in surface wettability, surface energy, and color. Our findings demonstrate that the dosage of argon ion implantation and the filler used have a profound impact on the properties of the modified surfaces. In general, ion implantation enhances the surface wettability of composites and pure PLA, with the recorded relationships being more pronounced in composites containing higher proportions of lignocellulosic fillers. Furthermore, the implantation of ions on the surface of composites induces changes in their color, opening up new possibilities for the field of materials science.

## 1. Introduction

Wood–plastic composites (WPC) are an effective combination of wood and plastics that are becoming increasingly popular in various industries such as construction, furniture, and packaging [1]. On the one hand, these composites behave like wood, but due to the thermoplastic matrix, they show properties different from wood; namely, they are resistant to moisture and biological corrosion, characterized by higher stiffness and lower linear shrinkage [2,3]. Different proportions of lignocellulosic particles and different types of thermoplastics used as components of WPC not only guarantee specific mechanical and physical properties but also determine certain aesthetic qualities. Scientific studies indicate that the content of lignocellulosic particles in WPC can be as high as 90% [4]. The most common types of plastics used as the matrix are polyethylene and polypropylene. However, natural and biodegradable polymers such as polylactide (PLA) or starch are being introduced more and more often [5].

As a plant-based material, PLA (polylactic acid) has a low environmental impact, making it an ideal candidate for creating biodegradable and environmentally friendly products. When combined with lignocellulosic particles, PLA has great potential for developing innovative composite materials that can support sustainable development and reduce environmental harm. Lignocellulosic particles, derived from plant biomass such as wood, serve as excellent raw materials for PLA composites. These natural fibers are rich in cellulose, hemicellulose, and lignin, which contribute to their high strength and stiffness [6]. Adding these natural fibers to PLA can significantly improve the mechanical properties of the composite, increasing its stiffness and tensile strength, and reducing its thermal expansion [7].

In addition, lignocellulosic particles contribute to the faster decomposition of composites in the environment, which is important in sustainability and plastic waste reduction. Compared to traditional plastics, PLA–lignocellulose composites have a much lower environmental impact.

However, to optimize the properties of WPC composites, it is necessary to properly prepare the raw materials or use the appropriate processing technology, such as extrusion or injection molding. Proper selection of PLA and lignocellulosic particle proportions is key to obtaining the final product’s desired mechanical and aesthetic properties [8]. However, even though these parameters can be insufficient, various studies have been undertaken to modify the properties of WPC [9,10]. One potential method for modifying the post-surface properties of WPC composites may be ion implantation [11,12]. Ion implantation, i.e., the introduction of atoms of any kind onto a solid after ionizing them and accelerating them in an electric field, is a well-known method for introducing changes in the microstructure, phase composition, and chemical composition of the modified surface layers of materials, especially metal alloys, silica, or glass [13,14].

The implantation of ions on the surface of WPC composites, a rare but potentially transformative topic in the scientific literature, presents a rich field for future research. More results on this topic can be found in the context of modifying plastics themselves [15]. Laput et al. [11] implanted a composite of polylactide and hydroxyapatite, which improved the material’s hydrophobic properties and showed that the implantation process leads to carbonization of the PLA surface layer. In turn, Tsuji et al. [16] increased the wettability of polystyrene and polydimethylsiloxane by implanting the surfaces of the polymers with negative carbon ions, which was not achieved for polylactide. On the other hand, Park et al. [17] produced a moderate hydrophilic PLA surface by demonstrating plasma immersion ion implantation in combination with conventional magnetron sputtering.

Ion implantation is a known method used in tribological and functional applications. For example, it is an important technique to improve the surface hardness and wear resistance of various materials or tools to change the mechanical, electrical, optical, and chemical properties of the implanted materials without changing the material surface and without creating an additional surface layer (no delamination). The ion implantation processes can also create new surface alloys instead of the bulk material or generate radiation damage instead of radiation. Such subtle applications are not possible using other plasma or chemical coating methods.

This article attempts to evaluate the application of the argon ion implantation method, which is a proven method in modifying the surface properties of metals and their alloys, but also plastics, to modify the post-surface properties of composites based on lignocellulosic particles in a PLA matrix. The applied method of improving the properties of WPC composites has not been analyzed in depth to date, as evidenced by the small body of scientific literature on the subject. However, this topic requires deeper analysis because of its potential for precisely modifying WPC materials to obtain the expected, sometimes functional, surface properties.

Ionic techniques, such as ion implantation in materials engineering, have advantages and disadvantages. The latter certainly includes process and equipment costs. Therefore, surface modification methods such as ion implantation would significantly increase production costs for the mass production of WPC composites. However, there is justification for using ion-implantation techniques on the surface of WPC composites, especially when they provide unique or functional properties, enabling them to acquire specific functions in specific applications.

## 2. Results and Discussion

### 2.1. Visual Characteristics of the Fabricated Composite

**Figure 1:**
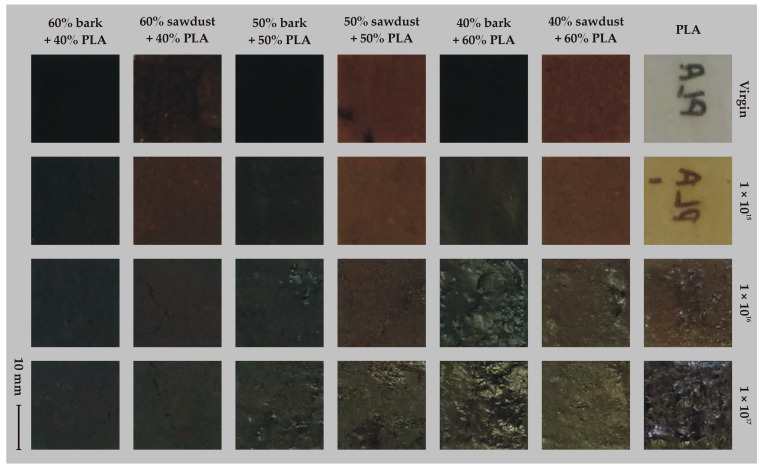
Visual characteristics of the WPC composite surfaces.

Figure 1 shows the visual surface characteristics of the implanted and control samples, highlighting the novelty and uniqueness of our research. The PLA samples, without lignocellulose doping, are semi-transparent, with a smooth surface and milky white color. The implantation of ions with a beam fluence of 1 × 10^15^ cm^−2^ causes the surface of the samples to remain semi-transparent but have an altered color. Larger values of ion fluence cause an apparent change in the color of the surface of the samples and their deformation. In the case of samples containing lignocellulosic particles, a similar trend in color changes is noticeable, but not as intense as in the control. An ion fluence of 1 × 10^15^ cm^−2^ does not cause apparent visual differences in surface quality compared to non-implanted samples, while higher doses of ion fluence cause a clear surface-darkening. In addition, it was found that with a higher proportion of PLA in the composite, the surface of the samples was more deformed. It was also noted in visual inspection that the type of cellulose particles determines the quality of the implanted surface. Based on visual signs, it was found that the surface of implanted composites containing bark particles was more deformed than the surface of composites containing wood particles. This phenomenon can be attributed to the chemical composition of the bark and wood, as well as to the geometry of the particles and their interaction in the PLA structure [18].

### 2.2. Modeling and Ion Implantation Results

**Figure 2:**
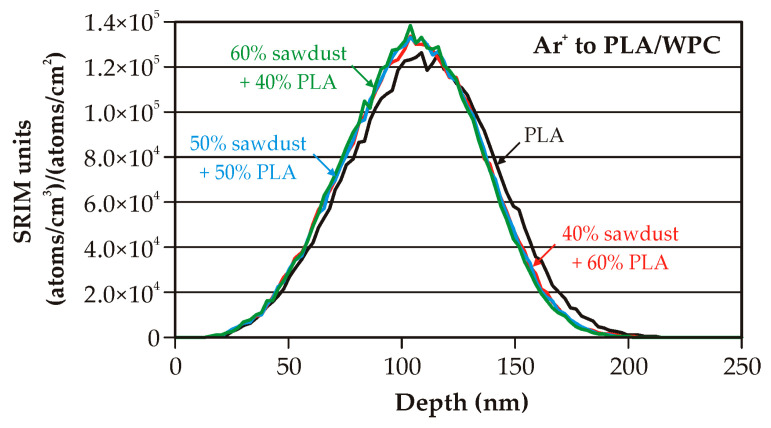
The modeled depth profiles for pure polylactide and three kinds of wood–plastic composite.

The results of the computer modeling of the depth profiles for argon ions implanted on four different substrates are presented in Figure 2. The substrates were as follows: pure PLA, 40% sawdust + 60% PLA, 50% sawdust + 50% PLA, and 60% sawdust + 40% PLA.

Table 1 shows the values of SRIM_max_ and associated peak volume dopant concentration, *N_max_*, for the fluence of 1 × 10^15^, 1 × 10^16^ and 1 × 10^17^ cm^−2^, projected range *R_p_*, range straggling Δ*R_p_*, skewness, kurtosis, total sputtering yield *Y_total_*, and sputtering yield for the four main elements of the substrates. The percentage values of relative difference from PLA are presented in round brackets.

The presented “SRIM units” in (atoms/cm^3^)/(atoms/cm^2^) are special units of plot ordinate used in SRIM code results. With these units multiplied by the ion fluence (in atoms/cm^2^), the ordinate values convert directly into a density distribution with the unit of atoms/cm^3^.

The profiles for all the WPCs are practically the same. This situation is different from WPC composites with a PE matrix, presented in our previous paper [19], due to the change in the density of the implanted substrate material. The measured and applied modeling density value was from 1.146 to 1.152 g/cm^3^ (change of about 0.5%) for the WPC with the PLA matrix, while the measured and applied density value was from 0.977 to 1.1 g/cm^3^ (change of about 12%) for the WPC with the PE matrix.

The differences in the values of the main parameters (maximum SRIM units, peak volume dopant concentration, projected range, range straggling) concerning the profile for pure PLA are at a few percent. However, the nature of the skewness of the implanted element profile changes, in the case of WPC, from positive to negative. The change in value is at the level of 2000% for each WPC. The change in kurtosis value is below 1% in each WPC case. The total modeled values of sputtering yield change are in the 4.18–7.55% range.

### 2.3. Results of Changes in Surface Wettability and Surface Free Energy

**Figure 3:**
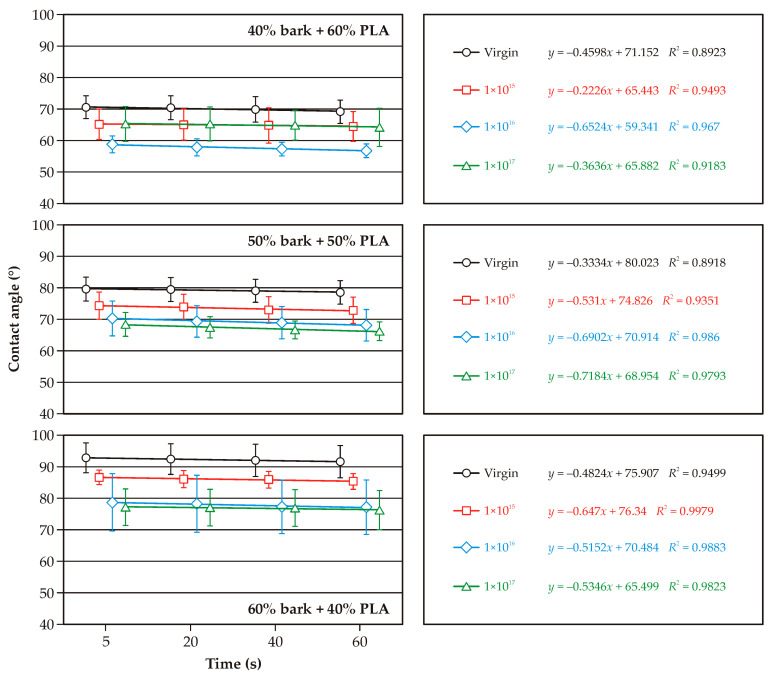
Variations in the surface wetting angles of PLA and bark-based WPC composites with ion implantation.

**Figure 4:**
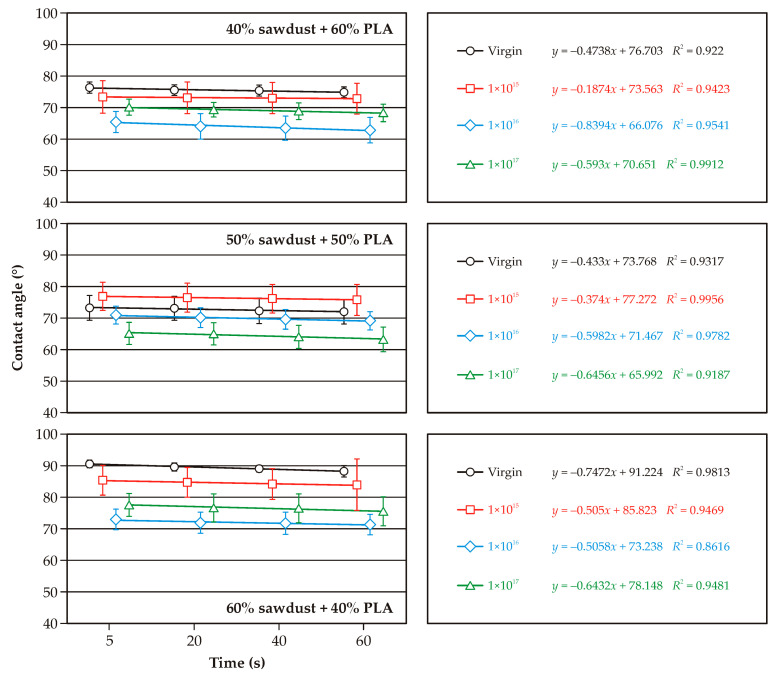
Variations in the surface wetting angle of PLA and sawdust-based WPC composites with ion implantation.

**Figure 5:**
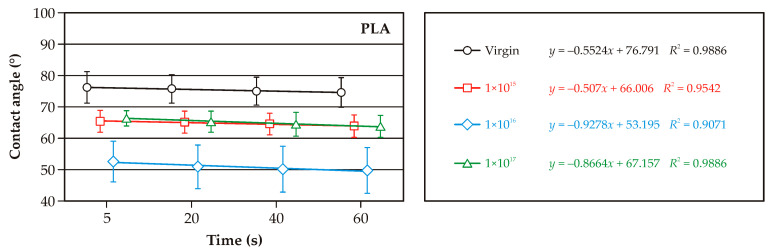
Variations in the surface wetting angle of PLA with ion implantation.

Figure 3, Figure 4 and Figure 5 illustrate the changes in wetting angle in WPC composites following argon ion implantation. The majority of cases show a positive effect, increasing the surface’s wettability. This finding holds significant potential for additional modification or finishing of WPC surfaces, offering the possibility of tailoring specific properties. It’s crucial to note the differences in wettability of the non-implanted samples. Composites with 60% added bark or sawdust exhibited a higher wetting angle and lower wettability than those with 40% lignocellulosic raw material (Figure 3 and Figure 4). This wettability was also lower than non-implanted PLA (Figure 5). As the content of lignocellulosic particles decreased, lower surface wettability was observed. The wetting angle of pure PLA was less than 80°, consistent with the literature data [20]. Both pure PLA and PLA–bark/sawdust composites absorb water, as evidenced by a decrease in the wetting angle at 60 s.

The smallest decrease in wetting angle over the observed time was found on the surface of the composites containing 40% bark. The implantation of argon ions on the surface of the composites caused a decrease in the wetting angle compared to the initial sample, which is evident in all the analyzed cases. It should be noted, however, that the ions’ fullness affects the changes in the wetting angle. In most of the analyzed cases, statistically significant differences were observed in the wettability of the surfaces of composites implanted with ion fluences of 1 × 10^15^ cm^−2^ and 1 × 10^17^ cm^−2^. Differences in the wettability of surfaces implanted with argon ions with fluences of 1 × 10^16^ cm^−2^ and 1 × 10^17^ cm^−2^ are somewhat challenging to determine as statistically significant, both for pure PLA and WPC composites. In the case of pure PLA, statistically significant differences were observed between the wetting angle of surfaces implanted with argon ions at a fluence of 1 × 10^17^ cm^−2^ and the other test variants. The observed decrease in the wetting angle in the samples implanted with argon ions indicates changes in the composites’ near-surface layers [21]. Our findings are further supported by the studies of Naoko et al. [22] and Vandemani [23], providing a robust foundation for our research. Also, acceleration energies can, but in a lesser way than ion fluence, affect changes in surface wettability [24]. Studies of nitrogen ion implantation on polycarbonate surfaces by Guzman et al. [25] showed that an increase in ion fluence is associated with increased surface degradation, which was also observed in the present study. Surface degradation or deformation can, in turn, affect changes in the wettability of WPC composites. Implantation of ions can also change the chemical composition of polymers and the formation of chemical groups that will react more readily with water [26].

Table 2 shows the surface free energy results obtained for composites and pure PLA implanted with argon ions. As can be easily seen, implantation causes an increase in surface free energy. The highest values were recorded for PLA samples implanted with ions with a fluence of 1 × 10^16^ cm^−2^. In the case of composites containing barium, the increase in surface energy was more pronounced in the samples containing a smaller amount of plant particles. In the case of WPC composites, it was also observed that the surface energy was higher on the surface of samples implanted with ions of higher fluence (1 × 10^16^ cm^−2^ and 1 × 10^17^ cm^−2^). It seems that the filler content in the form of sawdust or bark had the least effect on changes in the value of the surface free energy. However, the differences are marked by observing the results for composites containing as much as 60% lignocellulosic particles. Surface free energy is crucial to understanding surface phenomena mechanisms. The surface energy and the wetting angle determine the surface’s susceptibility to bonding and the adhesion of coatings [27]. For plastics such as PLA, the surface free energy is less than 40 mJ/m^2^, while modification of materials with an ion beam or plasma can increase the value of surface free energy [28].

### 2.4. Results of the Influence of Implantation on Surface Color Changes

Implantation of ions on the near-surface layers of the tested materials causes a change in surface color, which is visible to the observer, as evidenced by the values ΔE > 5. The surface of pure PLA also undergoes a significant color change. The differences in the color of the surface of pure PLA, subjected and not subjected to implantation, are more significant than the differences in the color of composites containing lignocellulose particles. For PLA composites containing 40 and 50% sawdust additive and whose surfaces were implanted with argon ions at a fluence of 1 × 10^15^ cm^−2^, no difference in color change visible to the observer’s eye was observed (ΔE < 5). Implantation of ions on the surface of PLA–bark/sawdust composites caused changes in the color of the surface compared to the original composite; at the same time, the differences in the observed color change between composites implanted with argon ions, especially for fluences of 1 × 10^16^ cm^−2^ and 1 × 10^17^ cm^−2^, were not visible to the observer’s eye.

The study also made it possible to observe a change in color balance between the various composites due to ion implantation. This is especially evident in changes in the brightness of the samples (L*) (Table 3). In addition, it should be noted that ion implantation generates color changes on the surface of samples containing bark and sawdust as filler in the direction of shades of yellow and green.

### 2.5. Statistical Analysis of Results

Table 4 presents an analysis of the percentage impact of individual variables (filler type, filler amount, ionizing particle amount, time after droplet placement) and their interactions on surface wetting angles. It is important to note that, except for filler type, all other factors showed a statistically significant effect (*p* < 0.05). On the other hand, interactions between these factors, in most cases (except for A × B, A × C, B × C, and A × B × C), did not have a statistically significant impact (*p* > 0.05) on the wetting angle values. Overall, it can be concluded that among the variables analyzed, filler amount (X = 43.27%) and ionizing particle number (X = 28.58%) had the most frequent percentage impact on the wetting angle. These were the dominant factors. In contrast, the percentage effect of the interaction between the amount of filler and the amount of ionizing particles was 8.03%. In comparison, the effect of the interaction between the filler type and the filler amount was only 2.66%. It is worth noting that the percentage effect of factors not included in the study was much higher, with Error = 15.82%.

## 3. Materials and Methods

### 3.1. Preparation of Composites

The raw materials for the composites were polylactic acid (PLA; Ingeo TM Biopolymer 2003D, NatureWorks LLC, Minnetonka, MN, USA) and lignocellulosic material in the form of sawdust and conifer bark. The moisture content of the lignocellulosic material was 5%. To produce the composites, particles passing through a sieve with a mesh size of 0.49 mm were used. The production of composites consisted of two stages:

(1) producing granules of the appropriate formulation using an extruder (Leistritz Extrusionstechnik GmbH, Nürnberg, Germany). The extrusion process took place at a temperature of 170–180 °C, and the resulting continuous strip of the composite was ground using a hammer mill.
(2) producing a composite of 300 mm × 300 mm × 0.5 mm in the process of flat pressing. The pressing was carried out in a single-shelf press (AB AK Eriksson, Mariannelund, Sweden) with the following parameters: pressing temperature 200 °C, maximum unit pressing pressure p_max_ = 1.25 MPa, and pressing time 6 min.

After pressing, the composite was cooled in the mold for 6 min in a cold press and then conditioned for 7 days under standard conditions (20 ± 2 °C; humidity 65 ± 5%). Three types of composites were produced, differing in the proportion of lignocellulosic particles (Table 5).

### 3.2. Ion Implantation and Modeling

The real ion implantation processes were meticulously preceded by the simulations/modeling. The depth profiles of the implanted argon and their main parameters were modeled using freeware type code SRIM-2013.00 (The Stopping and Range of Ions in Matter), (SRIM), in “Monolayer Collision Steps/Surface Sputtering” mode. The following parameters: *SRIM_max_*—maximum SRIM unit, *N_max_*—associated with its peak volume dopant concentration, *R_p_*—projected range, Δ*R_p_*—range straggling, skewness, kurtosis, and *Y*—sputtering yield [29,30], were determined with utmost precision.

The modeling also allows for determining the value of the sputtering yield. However, this phenomenon in substrate sputtering by the implanted ions does not account for this phenomenon, which is similar to the substrate damage and the chemical reactions between the implanted ions and/or the substrate components.

The modeling processes were performed with a comprehensive approach, simulating the behavior of 100,000 implanted ions perpendicular to the implanted substrate (the ion incidence angle was defined as 0°), due to the best conditions for the ion implantation. With the increase in the angle, the profile shifts towards the origin of the coordinate system; therefore, the value of the projected range *R_p_* decreases, the maximum volume concentration of the implanted element *N_max_* also decreases, and the range of straggling *ΔR_p_* also decreases. At the same time, we observe an increase in kurtosis and skewness towards the right [31]. The simulations were performed at absolute zero for all the cases.

The estimated (quantities of the elements) and measured (densities) values of the parameters for four types of implanted substrates were used. They were pure polylactide substrate material and three kinds of wood–plastic composites WPC, i.e.,:

- 100% polylactide (PLA),
- 40% sawdust + 60% PLA,
- 50% sawdust + 50% PLA,
- 60% sawdust + 40% PLA.

The weight percentages of the elements in pure PLA are about 51.28 wt.% of carbon, 2.95 wt.% of hydrogen, and 45.76 wt.% of oxygen, i.e., about 42.44 at.% of carbon, 29.13 at.% of hydrogen, and 28.43 at.% of oxygen, used in the modeling.

The chemical composition of wood varies from species to species; however, four elements, i.e., carbon, hydrogen, oxygen, and nitrogen, constitute the central mass of wood. Typically, dry wood contains about 49.6% carbon, 6.3% hydrogen, and 44.2% oxygen and nitrogen, by weight. The nitrogen content in wood is at the level of 0.12%. The content of other elements, e.g., calcium, potassium, sodium, magnesium, iron, manganese, sulfur, chlorine, silicon, and phosphorus, is about 1% by weight [32,33].

For the modeling, a selection of four (mentioned above) main elements, i.e., carbon, hydrogen, oxygen, and nitrogen, was used. Their total composition was standardized to 100% by weight in the first step. In the next step, the weight percentages were converted to atomic percentages used by the modeling code.

Finally, the chemical compositions of the WPC were determined from simple formulas:

(1) $${\%}_{X}=0.4{\%}_{Xsawdust}+0.6{\%}_{XPLA}\;\mathrm{for}\;\mathrm{WPC}\;\mathrm{of}\;40\%\;\mathrm{sawdust}+60\%\;\mathrm{PLA}$$

(2) $${\%}_{X}=0.5{\%}_{Xsawdust}+0.5{\%}_{XPLA}\;\mathrm{for}\;\mathrm{WPC}\;\mathrm{of}\;50\%\;\mathrm{sawdust}+50\%\;\mathrm{PLA}$$

(3) $${\%}_{X}=0.6{\%}_{Xsawdust}+0.4{\%}_{XPLA}\;\mathrm{for}\;\mathrm{WPC}\;\mathrm{of}\;60\%\;\mathrm{sawdust}+40\%\;\mathrm{PLA}$$

where ${\%}_{X}$—total content of element X in WPC, ${\%}_{Xsawdust}$—content of element X in sawdust, and ${\%}_{XPLA}$—content of element X in polylactide.

The modeling was performed only for sawdust reinforcement because the difference between the principal elements’ content in the sawdust and the bark is negligibly tiny [19,34].

The above-discussed final values of the elements’ content used in the modeling are listed in Table 6. These values are rounded to one or two decimal places, Additionally, the fundamental values of the substrate density have been shown. These values are rounded to three decimal places.

Argon, a carefully chosen element for implantation, was used in our experiments. The acceleration voltage was precisely set at 60 kV in all cases. This precision is crucial, as the values of the ion kinetic energy are numerically identical to the values of the accelerating voltage, i.e., 60 kV and 60 keV, due to the single ionization of the used ions.

Our experimental design was thorough, with the proposed fluencies of the implanted ions set at 1 × 10^15^, 1 × 10^16^, and 1 × 10^17^ cm^−2^ for all the substrate cases. This comprehensive approach ensures the validity of our findings.

The ion implantation processes of argon were provided using a semi-industrial, non-mass-separated implanter of gaseous ions with the continuous ion beam exploited in the National Centre for Nuclear Research Świerk in Otwock, Poland.

The implanted fluencies of argon were 1 × 10^15^, 1 × 10^16^, and 1 × 10^17^ cm^−2^. The acceleration voltage was 60 kV. The ion beam current was about 300 µA. Argon of 99.999% purity was used to source the implanted gaseous ions. The values of the process parameters were selected in the same way as in the case of PE ion implantation [18] to compare the obtained results directly.

The diameter of the ion beam of argon was about 4 cm. The dimensions of the implanted samples were about 20 × 20 × 3 mm^3^ for the sawdust reinforcement and about 20 × 20 × 2 mm^3^ for the bark reinforcement. Seven sets of samples, i.e., pure PLA, 40% sawdust + 60% PLA, 40% bark + 60% PLA, 50% sawdust + 50% PLA, 50% bark + 50% PLA, 60% sawdust + 40% PLA, and 60% bark + 40% PLA, were implanted on XY table for the decreasing of the temperature of samples surface (the estimated temperature value of the implanted samples should not exceed 150 °C) and the increasing of the implantation area.

Although ion implantation is a repeatable method, the investigation was conducted for typical sets of four samples.

### 3.3. Wetting Angle Measurement and Surface Free Energy

To determine the surface wetting angle of the composites, a Haas Phoenix 300 goniometer (Surface Electro Optics, Suwon City, Republic of Korea) was used. Measurements were taken for a polar liquid (distilled water) and a non-polar liquid (diiodomethane). The goniometer was equipped with a lens and a digital camera, and a droplet dispenser. Measurements were made at 5, 20, 40, and 60 s after the droplet (about 5 µL) was placed on the sample surface. Image XP analysis software (Surface Electro Optics, version 5.8, Suwon City, Republic of Korea) was used to analyze the droplet images and calculate the wetting angles. Each measurement was repeated in five replicates, both for the distilled water and the diiodomethane. The surface free energy of the samples was calculated for the average angle values using the Owens–Wendt method, which uses wetting angles obtained for both water and diiodomethane.

### 3.4. Assessment of the Surface Color of the Composites

A Datacolor ColorReaderPRO spectrophotometer (Datacolor Technology, Suzhou, China) was used to determine the color parameters of the samples before and after the ion implantation process. The acquired data was exported using the ColorReader App (Datacolor, Inc., Lawrenceville, NJ, USA). For each flat surface of the composite, 10 measurements were taken. The parameters were determined: L* (brightness), a* (chromatic coordinate on the red–green axis), and b* (chromatic coordinate on the yellow–blue axis). The total color difference (ΔE) was determined according to ISO 7724-3:2003 [35]. The absolute color difference (ΔE) was calculated to evaluate the color change that occurs on the surface of the composites before and after the implantation process. The interpretation of the results was based on the following criteria:

0 < ΔE ≤ 1—unnoticeable difference
1 < ΔE ≤ 2—difference noticed by an experienced observer
2 < ΔE ≤ 3.5—difference noticed by an inexperienced observer
3.5 < ΔE ≤ 5—noticeable difference
5 < ΔE—significant color change

### 3.5. Statistical Analysis

Statistical analysis of the results was performed using Statistica, version 13 (TIBCO Software Inc., Palo Alto, CA, USA). An analysis of variance (MANOVA) was used to test (significance level = 0.05) for significant differences between factors. A comparison of means was performed using the Tukey test, with a significance level of 0.05.

## 4. Conclusions

Implantation of argon ions on the surface of WPC composites containing PLA sawdust and bark caused changes in the surface properties of the materials. It was noted that the described changes depend on the fluence of the ions and the filler used for WPC production. Implantation of argon ions leads to a decrease in the contact angle and an increase in the surface free energy of both WPC and pure PLA. However, the changes found were more significant in those materials that contained a higher proportion of lignocellulosic particles. The implantation of ions on the surface of WPC also causes changes in the coloration of the composites. In addition, higher doses of ion fluence worsen the quality of the surface.

Ion implantation is an effective method in imparting new surface properties to WPC, which can play an important role in composite surface finishing, bonding, painting, or laminating. With suitably adjusted implantation parameters, the adhesive properties of composites can be modified in a targeted manner for further applications and transfer of WPC composites. Therefore, ion implantation on the surface of WPC is a topic worthy of further research and design work.

## Figures and Tables

**Table 1:** Peak parameter modeling values of ions implanted on the surface of composites.

Parameter	PLA	40% Sawdust + 60% PLA	50% Sawdust + 50% PLA	60% Sawdust + 40% PLA
*SRIM_max_*	1.26 × 10^5^	1.34 × 10^5^	1.33 × 10^5^	1.38 × 10^5^
(atoms/cm^3^)/(atoms/cm^2^)		(5.99%)	(5.51%)	(9.63%)
*N_max_* (cm^−3^)	1.26 × 10^20^	1.34 × 10^20^	1.33 × 10^20^	1.38 × 10^20^
		(5.99%)	(5.51%)	(9.63%)
	1.26 × 10^21^	1.34 × 10^21^	1.33 × 10^21^	1.38 × 10^21^
		(5.99%)	(5.51%)	(9.63%)
		1.347 × 10^22^	1.33 × 10^22^	1.38 × 10^22^
	1.26 × 10^22^	(5.99%)	(5.51%)	(9.63%)
*R_p_* (nm)	109	105.2	105.2	104.4
		(−3.49%)	(−3.49%)	(−4.22%)
Δ*R_p_* (nm)	62	59	58.6	57.8
		(−4.84%)	(−5.48%)	(−6.77%)
Skewness	0.0024	−0.0415	−0.0446	−0.0502
		(−1829.17%)	(−1958.33%)	(−2191.67%)
Kurtosis	2.7169	2.6974	2.7059	2.7073
		(−0.72%)	(−0.4%)	(−0.35%)
*Y_C_* (atoms/ion)	0.3558	0.3145	0.304	0.3006
		(−11.61%)	(−14.56%)	(−15.51%)
*Y_H_* (atoms/ion)	0.5627	0.7191	0.7606	0.809
		(27.79%)	(35.17%)	(43.77%)
*Y_O_* (atoms/ion)	0.5637	0.5098	0.4931	0.4837
		(−9.56%)	(−12.52%)	(−14.19%)
*Y_N_* (atoms/ion)		0.00078	0.00044	0.0008
*Y_total_* (atoms/ion)	1.4822	1.54418	1.55814	1.5941
		(4.18%)	(5.12%)	(7.55%)

**Table 2:** The values of surface free energy.

Filler Type	Filler Amount (%)	Fluence (cm^−2^)			
		0	1 × 10^15^	1 × 10^16^	1 × 10^17^
		Surface Free Energy (mJ/m^2^)			
Bark	0	46.3	52.4	58.2	51.9
	40	48.7	53.0	57.0	52.4
	50	42.7	47.5	49.6	50.4
	60	39.0	42.2	46.0	46.9
Sawdust	0	46.3	52.4	58.2	51.9
	40	47.3	49.1	52.5	51.5
	50	42.4	47.2	49.7	52.2
	60	46.3	50.5	54.3	51.9

**Table 3:** Evaluation of lab values on WPC surfaces before and after ion implantation.

Filler Type	Filler Amount (%)	Fluence (cm^−2^)	L*	a*	b*	ΔE
Bark	0	0	67.578	2.678	−4.500	
	40		23.760	1.357	1.342	
	50		22.743	1.083	1.378	
	60		24.870	1.565	1.448	
Sawdust	0	0	67.578	2.678	−4.500	
	40		38.800	9.125	19.356	
	50		38.086	9.535	18.375	
	60		30.488	4.481	8.529	
Bark	0	1 × 10^15^	58.565	2.679	9.309	16.490
	40		34.141	0.590	4.003	10.744
	50		31.180	1.326	2.724	8.547
	60		33.654	0.671	2.217	8.863
Sawdust	0	1 × 10^15^	58.565	2.679	9.309	16.490
	40		41.312	7.761	16.742	3.873
	50		42.763	8.080	16.757	5.158
	60		39.224	4.322	9.234	8.766
Bark	0	1 × 10^16^	44.992	3.226	1.371	23.343
	40		38.160	2.506	−0.736	14.594
	50		36.663	0.490	1.230	13.933
	60		38.546	0.346	0.891	13.742
Sawdust	0	1 × 10^16^	44.992	3.226	1.371	23.343
	40		40.552	3.509	4.350	16.118
	50		39.855	3.321	4.074	15.693
	60		40.659	1.760	2.050	12.362
Bark	0	1 × 10^17^	38.128	−0.910	1.564	30.281
	40		41.801	0.932	1.774	18.051
	50		40.439	0.925	2.715	17.747
	60		41.614	0.093	2.608	16.849
Sawdust	0	1 × 10^17^	38.128	−0.910	1.564	30.281
	40		41.408	1.730	4.290	16.984
	50		39.922	−1.998	7.614	15.880
	60		41.263	0.926	3.238	12.519

**Table 4:** Statistical analysis of the contact angle.

Factors	*p*	X (%)
Filler type (A)	1.57 × 10^−1^	0.06
Filler amount (B)	<1.00 × 10^−17^	43.27
Implanted fluencies of ions (C)	<1.00 × 10^−17^	28.58
Time after placing a droplet (D)	7.34 × 10^−3^	0.38
A × B	<1.00 × 10^−17^	2.66
A × C	1.08 × 10^−2^	0.35
B × C	<1.00 × 10^−17^	8.03
A × D	9.96 × 10^−1^	<0.01
B × D	1.00	0.01
C × D	1.00	0.02
A × B × C	2.87 × 10^−3^	0.79
A × B × D	1.00	<0.01
A × C × D	1.00	<0.01
B × C × D	1.00	0.01
A × B × C × D	1.00	0.01
Error		15.82

*p*—significant with α = 0.05; X—percentage of contribution.

**Table 5:** Characteristics of the composition of raw materials used for the production of WPC.

Samples	PLA (%)	Sawdust (%)	Bark (%)
40% bark + 60% PLA	60	-	40
40% sawdust + 60% PLA	60	40	-
50% bark + 50% PLA	50	-	50
50% sawdust + 50% PLA	50	50	-
60% bark + 40% PLA	40	-	60
60% sawdust + 40% PLA	40	60	-
PLA	100	-	-

**Table 6:** The values of the atomic percent and the densities of the substrates used in the modeling.

Substrate	at.% C	at.% H	at.% O	at.% N	Density (g/cm^3^)
PLA	42.44	29.13	28.43	-	1.134
40% sawdust + 60% PLA	38.03	36.5	25.44	0.03	1.152
50% sawdust + 50% PLA	36.93	38.34	24.7	0.03	1.146
60% sawdust + 40% PLA	35.83	40.18	23.95	0.04	1.148

## Data Availability

The original contributions presented in the study are included in the article; further inquiries can be directed to the authors.
